# Effect of Photoconversion Coatings for Greenhouses on Electrical Signal-Induced Resistance to Heat Stress of Tomato Plants

**DOI:** 10.3390/plants11020229

**Published:** 2022-01-17

**Authors:** Marina Grinberg, Ekaterina Gromova, Alyona Grishina, Ekaterina Berezina, Maria Ladeynova, Alexander V. Simakin, Vladimir Sukhov, Sergey V. Gudkov, Vladimir Vodeneev

**Affiliations:** 1 Department of Biophysics, Lobachevsky State University of Nizhny Novgorod, Gagarin St., 23, 603950 Nizhny Novgorod, Russia; mag1355@yandex.ru (M.G.); kater333@inbox.ru (E.G.); 79159532707@yandex.ru (A.G.); ladeynova.m@yandex.ru (M.L.); vssuh@mail.ru (V.S.); S_makariy@rambler.ru (S.V.G.); 2 Department of Biochemistry and Biotechnology, Lobachevsky State University of Nizhny Novgorod, Gagarin St., 23, 603950 Nizhny Novgorod, Russia; berezina.kat@gmail.com; 3 Prokhorov General Physics Institute of the Russian Academy of Sciences, Vavilova St., 38, 119991 Moscow, Russia; avsimakin@gmail.com

**Keywords:** photoconversion coatings, stress signals, photosynthesis, reactive oxygen species, resistance to environment stressors, *Solanum lycopersicum*

## Abstract

The use of photoconversion coatings is a promising approach to improving the quality of light when growing plants in greenhouses in low light conditions. In this work, we studied the effect of fluoropolymer coatings, which produce photoconversion of UV-A radiation and violet light into blue and red light, on the growth and resistance to heat stress of tomato plants (*Solanum lycopersicum* L.). The stimulating effect of the spectrum obtained as a result of photoconversion on plant growth and the activity of the photosynthesis process are shown. At the same time, the ability to withstand heat stress is reduced in plants grown under a photoconversion coating. Stress electrical signals, which normally increase resistance, in such plants have a much weaker protective effect on the photosynthetic apparatus. The observed effects are apparently explained by a decrease in the concentration of H_2_O_2_ in plants grown using photoconversion technologies, which leads to a shift in the development program towards increased productivity to the detriment of the protective function. Thus, when using photoconversion technologies in agricultural practice, it is necessary to pay increased attention to maintaining stable conditions during plant cultivation.

## 1. Introduction

Improving the efficiency of greenhouses is an urgent problem in agriculture. In temperate and polar latitudes, one of the most serious problems is insufficient illumination, in particular at wavelengths used in the course of photosynthetic reactions. This problem can be solved by active backlighting using LEDs or other light sources [1,2,3,4,5,6]. However, this approach requires high economic and energy costs, which leads to an increase in carbon footprint [7]. Another approach is passive spectrum correction using films and coatings. To date, research on photoactive coatings for greenhouses has focused mainly on three aspects: protection against near-infrared radiation, protection against UV radiation and the conversion of photons of photosynthetically inactive spectral ranges into blue and red light [8,9,10,11]. The latter approach seems especially promising, since it demonstrates a significant stimulating effect on morphometric parameters and the activity of physiological processes in various plant species [10,11,12,13,14,15].

In addition to productivity for plants, resistance to various stressful influences is of great importance. In greenhouse conditions, the most significant damaging factors for plants are high temperatures on sunny days and various pathogenic organisms. For the formation of complex resistance to stressors at the level of the whole plant, distant stress signals are important [16,17,18]. As a rule, the signal is generated in the most vulnerable or sensitive zone of the plant, for example, in the upper young leaves, and then spreads throughout the body, modulating intracellular signaling pathways and the activity of physiological processes, which leads to an increase in resistance [17,18]. Distant stress signals, depending on the nature, are subdivided into several types, each of which is induced by its own set of stressors and has a characteristic speed of propagation and scale of coverage. Hydraulic, chemical and electrical signals are distinguished [16,19,20]. Electrical signals (ES), representing a wave of changes in the electrical potential on cell membranes, are of particular interest under the action of rapidly growing stressors, to which it is required to quickly increase resistance at the level of the whole plant. ROS and Ca^2+^ signaling systems are involved in the generation and propagation of ES. Entering cells through, presumably, ROS-activated cation channels, Ca^2+^ causes the opening of anion channels and inactivation of proton ATPase; as a result of which an electrical signal develops [16,20]. Such signals are induced by a wide range of stimuli, including changes in temperature, humidity, excessive lighting and attack by leaf-eating insects [21]. Spreading through the plant at a rate of the order of mm × s^−1^, ES cause functional responses, first of all, a temporary inhibition of the light-driven and light-independent reactions of photosynthesis and transpiration, which allows plants to survive an unfavorable period with minimal damage [22,23]. It should be noted that the ROS and Ca^2+^ systems participating in long-distance signaling are modulated by lighting conditions [21,24,25].

In our previous works [10,14], we showed that fluoropolymer coatings, which produce photoconversion of UV-A radiation and violet light into blue and red light, stimulate the growth, development and yield of plants but inhibit heat-induced electrical signals. In this work, we study the effect of these photoconversion coatings on the electrical signal-induced resistance of tomato plants to heat stress.

## 2. Materials and Methods

### 2.1. Growing Plants under Photoconversion Coating

The studies were carried out on 5-week-old tomato plants (*Solanum lycopersicum* L.), variety “Komnatnyj syurpriz” (undersized, early maturing). The plants were grown in a vegetation room under a 16-h light period at a constant air temperature of 23 °C and a relative humidity of about 50%. Plants were grown in individual pots in growing media (peat 96%, dolomite flour 4%; pH = 5.8), watering was carried out two times a week.

**Figure 1:**
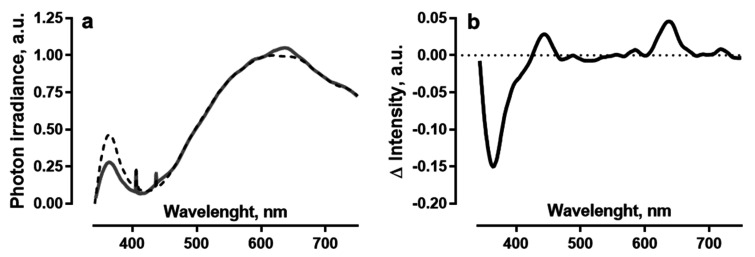
(**a**) Normalized photoluminescence spectrum of a fluoropolymer film containing fluorophores and gold nanoparticles (solid line) and without photoconverting components (dotted line); (**b**) the difference between the intensities of the spectra of a fluoropolymer photoconversion film and a fluoropolymer equalizing filter.

A combination of incandescent (95 W; Brest Electric Lamp Plant, Brest, Belarus) and UV lamps (Litarc Lighting and Electronic Ltd., Shenzhen, China; maximum emission at 365 nm) was used as a light source to simulate solar light. Photoconversion coatings were placed between the lamps and plants. For the photoconversion coating, we used fluoropolymer films containing gold nanoparticles and Cd_(1−x)_Zn_(x)_Se quantum dots [10], located between plants and light sources. Films produce photoconversion of UV-A radiation and violet light into blue (photoluminescence maximum 450 nm) and red light (photoluminescence maximum 630 nm). When using a light-converting composite film, we have light close to the emission spectrum of a blackbody with the imposition of “blue” and “red” components on it (Figure 1). The photoluminescence spectrum of the photoconversion coating obtained using a broader spectrum xenon reference light source is shown in Figure S1 in the Supplementary Materials. The photoluminescence maxima of photoconversion films obtained upon irradiation with a combination of light sources used in the experiment correspond to the maxima obtained upon irradiation with a reference source (Figure 1 and Figure S1). The light intensity at the plant leaf level was 247 μmol photons m^−2^ s^−1^. Control plants were grown under similar conditions using a luminous flux equalization filter (fluoropolymer films without photoconverting components) instead of a photoconversion coating. The spectrum of the light sources after the neutral filter corresponded to the spectrum of the light sources itself. The proportion of UV-A radiation (measured from 340 to 400 nm) was 7.8% in the total spectrum (from 340 to 750 nm), which is close to the distribution of light energy in the spectrum of solar radiation (UV 6%, visible light 52% and IR 42%) [26]. Spectrum parameters were recorded using an Ocean Insight FLAME spectrometer (Ocean Insight, Largo, FL, USA) and a PM100D radiation power/energy meter complex (Thorlabs, Newton, NJ, USA).

### 2.2. Methods

#### 2.2.1. Measurement of Morphometric Indicators

The growth rate of plants was estimated from the leaf area. The analysis of the total leaf area of individual plants was carried out from photographs using a macro based on the ImageJ program. Photographing was carried out every 3–5 days throughout the entire period of plant cultivation.

#### 2.2.2. Measurement of the Parameters of Photosynthesis and Transpiration

To register the parameters of photosynthesis and transpiration, a GFS-3000 infrared gas analyzer and a Dual-PAM-100 PAM fluorometer with a Dual-PAM gas-exchange Cuvette 3010-Dual measuring head (Heinz Walz GmbH, Pfullingen, Germany) were used. The calculation of the efficiency of PSII photochemistry (F_v_/F_m_), the quantum yield of photochemical reactions of photosystem I (Φ_PSI_) and photosystem II (Φ_PSII_) and non-photochemical quenching of fluorescence (NPQ) were carried out in accordance with [27,28]:

- F_m_ is the maximum yield of fluorescence after dark adaptation;
- F_v_ = F_m_ − F_0_, where F_0_ is level of fluorescence without light and after dark adaptation;
- Φ_PSI_ = (P_m_′ − P)/P_m_, where P_m_ is the maximum change in P_700_ signal of photosystem I, which reflects the complete oxidation of P_700_, P_m_′ is the maximum change in P_700_ signal in the light and P is the current level of P_700_ signal;
- Φ_PSII_ = (F_m_′ − F_t_)/F_m_′, where F_m_′ is the maximum yield of fluorescence in the light and F_t_ is the steady-state value of fluorescence immediately prior to the flash;
- NPQ = (F_m_ − F_m_′)/F_m_′.

To maintain photosynthesis (actinic light), illumination with a wavelength of 460 nm was used; the photon flux density was 239 μmol × m^−2^ × s^−1^. For saturating light pulses, illumination at a wavelength of 635 nm with a photon flux density of 10,000 μmol m^−2^ × s^−1^ was used. The duration of saturating light pulses was 300 ms and the interval between flashes was 10 s. The concentration of CO_2_ in the measuring cell was maintained at 360 μmol × mol^−1^, temperature 23 °C, relative humidity 60%. The area of the investigated area of the leaf was 1.3 cm^2^. Measurements were made on the second adult leaf from the top. The recording of the dark parameters of assimilation and transpiration began 10 min after plant fixation and continued for 15 min. Then, together with the gas exchange, registration of the light-driven reactions of photosynthesis began, which lasted 20 min.

#### 2.2.3. Registration of Long-Distance Electrical Signals

An electrical signal was induced by gradual heating of the tip of the upper adult leaf (2 cm^2^) in a cuvette with water heated to 55 °C. Electric potentials were recorded from the plant surface using Ag^+^/AgCl macroelectrodes. The measuring electrode was located on the petiole of the heated leaf. The contact of the electrode with the leaf was carried out through a thread soaked in a standard solution (1 mM KCl, 0.5 mM CaCl_2_ and 0.1 mM NaCl). The reference electrode was located in the soil. The potential difference was measured by a multichannel system for measuring biopotentials, Multitest IPL-113 (Semico, Russia), and processed on a PC using the Param 2 program. The adaptation between plant fixation and signal induction was 1 h.

#### 2.2.4. Measurement of Resistance to Heat Stress Induced by Electrical Signals

Before placing the plants in stressful conditions, the level of photosynthesis in them at rest was recorded and ES was induced. Heat stress was caused 45 min after ES by total heating of a vessel with plants in a TS-1/80 SPU thermostat (Smolensk SKTB SPU, Smolensk, Russia) for 45 min at a temperature of 46 °C. The plant resistance to heat stress was assessed by the level of residual photosynthesis 1 h after the end of heating.

#### 2.2.5. Determination of H_2_O_2_ Content

The change in hydrogen peroxide (H_2_O_2_) concentration in tomato leaves was analyzed using 3,3′-diaminobenzidine (DAB) staining. Intact middle-aged leaves were immersed in a staining solution (1 mg/mL DAB, 10 mM KH_2_PO_4_ and 0.05% *v/v* Tween 20) and infiltrated in a vacuum desiccator at −50 kPa for 1.5 min 3 times with pauses for 3 min. Incubation was carried out for 4 h in the dark on an OS-20 orbital shaker (BioSan, Riga, Latvia) at 100 rpm. The leaves were then decolorized with 96% ethanol by heating to 80 °C. Leaves were photographed using a stereomicroscope (Meiji Techno, Chikumazawa, Japan) and a digital camera. Image processing was performed in the ImageJ program. The color intensity was determined by the region of interest of a fixed value from each leaf.

#### 2.2.6. Statistics

The results were obtained on plants grown in two separate series: the first series from June to August and the second from August to October 2021. The number of plants in each series was 18–20. Each type of experiment comprised 6–12 repetitions; every replicate was performed on a separate plant. The mean and standard error of the mean were calculated and the normal data distribution was confirmed for all the experiments; the significance of differences was determined by the Student’s *t*-test. The results also show typical records of the parameters of photosynthesis and transpiration at rest and after propagation of an electrical signal, obtained in control plants.

## 3. Results

### 3.1. Effect of Photoconversion Coatings on Morphometric Parameters of Plants

**Figure 2:**
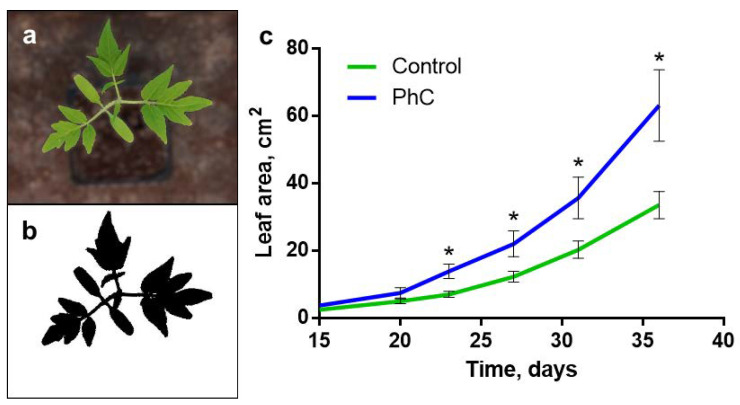
Influence of photoconversion coatings on the leaf area of tomato plants: (**a**,**b**) a photograph of a control tomato plant and a “mask” for calculating the leaf area using the ImageJ macro; (**c**) dynamics of changes in the leaf area of control plants and plants grown using the photoconversion coating (PhC). *: *p* < 0.05.

Cultivation under photoconversion coatings stimulates the growth of leaves of tomato plants (Figure 2). Differences in leaf area begin to appear in the third week—with the development of the first true (not cotyledonous) leaf. In the following weeks, differences in leaf area increase. On the 36th day, the leaf area of the control plants is 33 ± 4 cm^2^ and the leaf area of plants grown under photoconversion coatings is 63 ± 11 cm^2^.

### 3.2. Effect of Photoconversion Coatings on Photosynthetic Activity and Transpiration Rate

**Figure 3:**
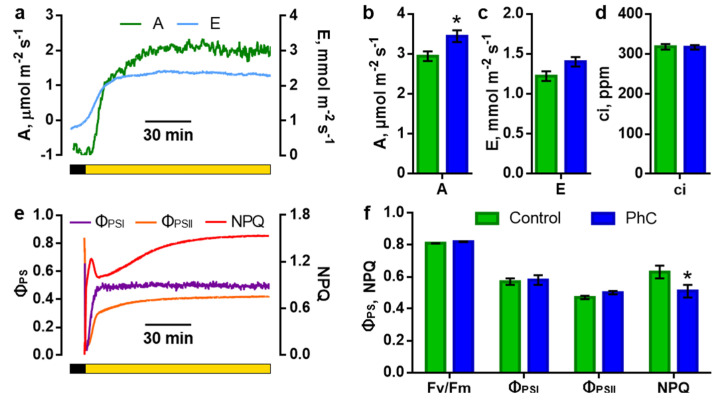
Influence of a photoconversion coating on the activity of photosynthesis and the intensity of transpiration of tomato plants: (**a**) a typical record of the responses of assimilation and transpiration of a control tomato plant to the switching on of the light; (**b**–**d**) the effect of the photoconversion coating on the level of assimilation, the intensity of transpiration and intercellular CO_2_ concentration; (**e**) a typical record of the light-driven reactions of photosynthesis of a control tomato plant to light switching on; (**f**) the effect of the photoconversion coating on the light-driven reactions of photosynthesis. A: assimilation of CO_2_; E: transpiration; ci: intercellular CO_2_ concentration; F_v_/F_m_: efficiency of PSII photochemistry; Φ_PSI_ and Φ_PSII_: quantum yield of photochemical reactions of photosystems I and II; NPQ: non-photochemical quenching of fluorescence; PhC: photoconversion. *: *p* < 0.05.

The effect of growing under a photoconversion coating on the physiological processes of tomato seedlings was assessed by the parameters of the light-driven reactions of photosynthesis (efficiency of PSII photochemistry (F_v_/F_m_), quantum yield of photochemical reactions of photosystem I (Φ_PSI_) and photosystem II (Φ_PSII_) and non-photochemical quenching of fluorescence (NPQ)) and indicators of gas exchange—the activity of assimilation of CO_2_ (A) and the intensity of transpiration (E) in the light. In tomato seedlings, the reaction to the switching on of light for all the studied parameters is a gradual transition from the dark level to the light level, reaching a plateau in about 20–30 min (Figure 3a,e).

Activation of photosynthesis was registered in tomato plants grown under a photoconversion coating. The level of the integral indicator of photosynthesis—CO_2_ assimilation—significantly increased from 2.94 ± 0.12 to 3.44 ± 0.15 μmol × m^−2^ × s^−1^ (Figure 3b). In this case, the intensity of transpiration did not undergo statistically significant changes and was 1.22 ± 0.06 mmol × m^−2^ × s^−1^ in control plants and 1.40 ± 0.06 mmol × m^−2^ × s^−1^ in those grown under a photoconversion coating (Figure 3c). Intercellular CO_2_ concentration (ci) also showed no dependence on the photoconversion coating: 318 ± 7 ppm in control plants and 317 ± 6 in plants grown using photoconversion coatings (Figure 3d). Analysis of the light-driven reactions showed a decrease in the NPQ level from 0.63 ± 0.04 to 0.51 ± 0.04 in plants grown using photoconversion coatings (Figure 3f). Indicators F_v_/F_m_ and Φ_PSII_ do not differ from control.

### 3.3. Effect of Photoconversion Coatings on Electrical Signal-Induced Heat Stress Tolerance

**Figure 4:**
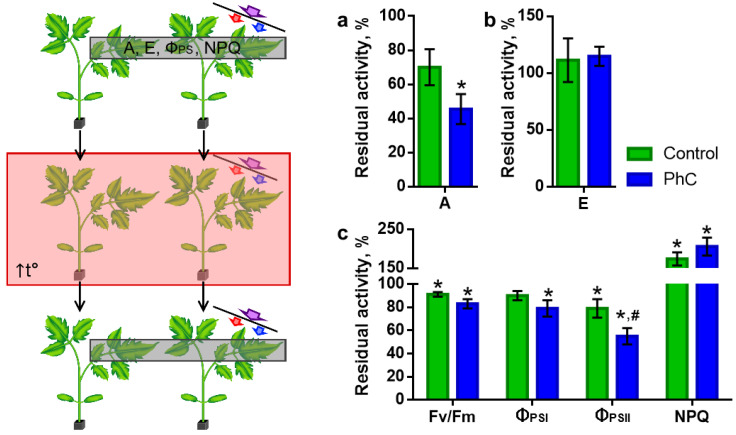
Effect of a photoconversion coating on the resistance of tomato plants to heat stress, which was determined by the residual activity of physiological processes after heating: (**a**) residual activity of assimilation; (**b**) residual activity of transpiration; (**c**) residual activity of light-driven photosynthesis reactions. Residual activity is expressed in percentages, where 100% corresponds to the level before HS. The measurement scheme is shown on the left. Photosynthesis levels were first recorded (gray rectangle in the scheme); then, plants were subjected to heat stress for 45 min at a temperature of 46 °C (red rectangle in the scheme). The residual levels of photosynthesis were recorded 1 h after heat stress. A: assimilation of CO_2_; E: transpiration; F_v_/F_m_: efficiency of PSII photochemistry; Φ_PSI_ and Φ_PSII_: quantum yield of photochemical reactions of photosystems I and II; NPQ: non-photochemical quenching of fluorescence; PhC: photoconversion. *: the difference between “after HS” and “before HS” is significant, *p* < 0.05; #: the difference between “control” and “photoconversion” is significant, *p* < 0.05.

To analyze the resistance of plants grown under the photoconversion coating to stressors, tomato seedlings were exposed to elevated temperatures. The effect of heat stress (HS) was assessed by the residual activity of photosynthesis (Figure 4).

After the action of heat stress, both in control plants and those grown using a photoconversion coating, a significant suppression of photosynthetic processes was observed (Figure 4). An hour after the end of heating in the control, the residual level of F_v_/F_m_ was 91 ± 2%, Φ_PSII_ was 90 ± 4%, Φ_PSI_ was 79 ± 8%, NPQ was 174 ± 2% and A was 70 ± 11%. In this case, the intensity of transpiration did not significantly differ from the control and was 111 ± 19%. For plants grown under photoconversion films, the residual level of photosynthetic parameters was 83 ± 4%, 79 ± 7%, 55 ± 7%, 206 ± 23% and 45 ± 9%, respectively. The intensity of transpiration was 115 ± 8%. Thus, plants grown using photoconversion technologies exhibit lower resistance to heat stress, which is indicated by a tendency towards a decrease in all photosynthetic parameters and a significantly lower level of residual Φ_PSII_ after heating.

**Figure 5:**
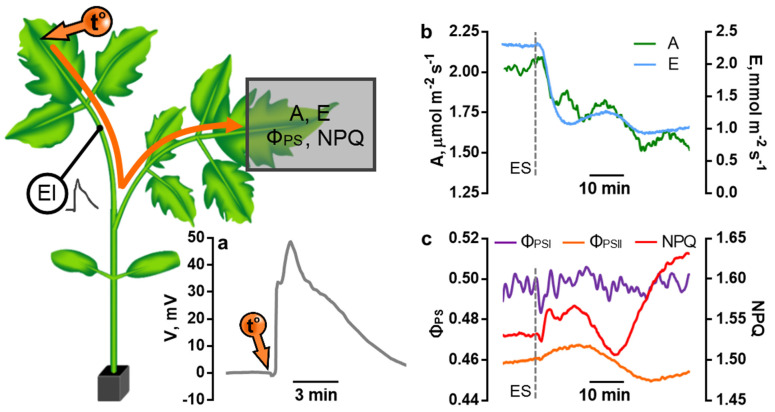
Local heating-induced electrical signal in a tomato plant: (**a**) Measurement scheme and typical record of an electrical signal of a control tomato plant. The electrical signal was induced by gradual heating of the tip of the upper adult leaf in a cuvette with water heated to 55 °C. Electric potentials were recorded with an electrode (El) on the petiole of the heated leaf. Photosynthesis parameters were recorded in the second from the top adult leaf. (**b**) The responses of the light-driven reactions of photosynthesis evoked by an electrical signal of a control tomato plant. (**c**) The responses of assimilation and transpiration evoked by an electrical signal of a control tomato plant. A: CO_2_ assimilation; E: transpiration; F_v_/F_m_: potential quantum yield; Φ_PSI_ and Φ_PSII_: quantum yield of photochemical reactions of photosystems I and II; NPQ: non-photochemical quenching of fluorescence.

An electrical signal induced by a local stimulus is a depolarization wave propagating from the stimulation zone with an amplitude of several tens of mV (Figure 5a). In tomato plants, an electrical signal induced by local heating leads to a transient change in the activity of both the light-driven and light-independent reactions of photosynthesis in the neighboring leaf: the level of A and Φ_PSI_ decreases and the level of NPQ increases (Figure 5b,c). The intensity of transpiration decreases as a result of signal transmission (Figure 5b).

**Figure 6:**
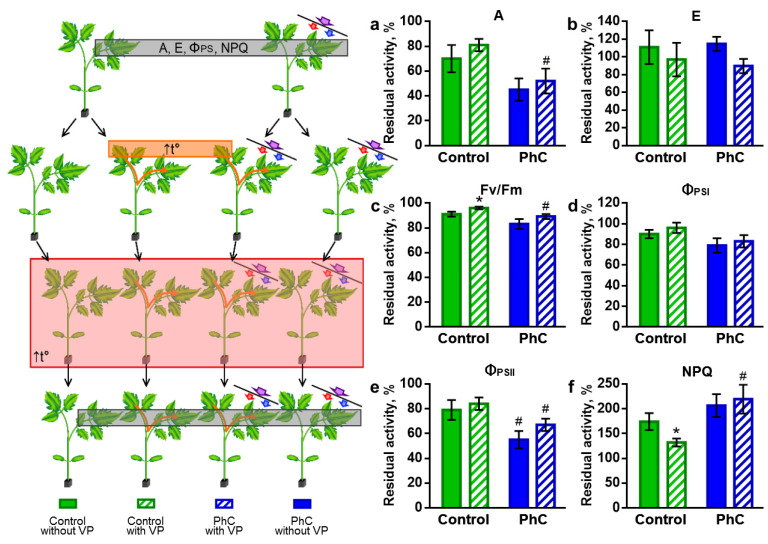
Influence of photoconversion coatings on electrical signal-induced resistance of tomato plants to heat stress, which was determined by the residual activity of physiological processes after heating: (**a**) residual activity of assimilation; (**b**) residual activity of transpiration; (**c**–**f**) residual activity of light-driven photosynthesis reactions. Residual activity is expressed in percentages, where 100% corresponds to the level before HS. The measurement scheme is shown on the left. Photosynthesis levels were first recorded (gray rectangle in the scheme); then, in half of the plants, ES was induced by short-term heating of the top leaf (orange rectangle and arrow in the scheme); after 45 min, all the plants were subjected to heat stress for 45 min at a temperature of 46 °C (pink rectangle in the scheme). The residual levels of photosynthesis were recorded 1 h after heat stress. A: assimilation of CO_2_; E: transpiration; F_v_/F_m_: efficiency of PSII photochemistry; Φ_PSI_ and Φ_PSII_: quantum yield of photochemical reactions of photosystems I and II; NPQ: non-photochemical quenching of fluorescence; PhC: photoconversion. *: the difference between “without ES” and “with ES” is significant, *p* < 0.05; #: the difference between “control” and “photoconversion” is significant, *p* < 0.05.

Electrical signals and the functional responses elicited by them contribute to an increase in plant resistance to stressors, including heat stress (Figure 6).

In tomato plants, in which heat-induced ES occurs before heat stress, higher levels of residual parameters of photosynthesis were recorded (Figure 6). A significant increase in the F_v_/F_m_ level was shown (96 ± 1% after ES instead of 91 ± 2% in the control) and a decrease in the NPQ level was observed (132 ± 8% after ES instead of 174 ± 17% in the control). ES had no effect on the level of transpiration. In plants grown using a photoconversion coating, there was only a tendency to an increase in the residual levels of indicators in the presence of ES, and no statistically significant differences were found. As a result, such plants after heat stress demonstrated significantly lower levels for a number of key parameters in comparison with the control with ES (F_v_/F_m_ was 96 ± 1% in the control and 89 ± 2% under the films, Φ_PSII_ was 84 ± 5% in control and 67 ± 5% under the films, NPQ was 132 ± 8% in the control and 219 ± 29% under the films, A was 81 ± 5% in the control and 52 ± 10% under the films). In general, stress ESs in plants grown under photoconversion films have reduced protective properties with respect to photosynthesis under heat stress.

### 3.4. Effect of Photoconversion Coatings on the H_2_O_2_ Content in Plant Leaves

**Figure 7:**
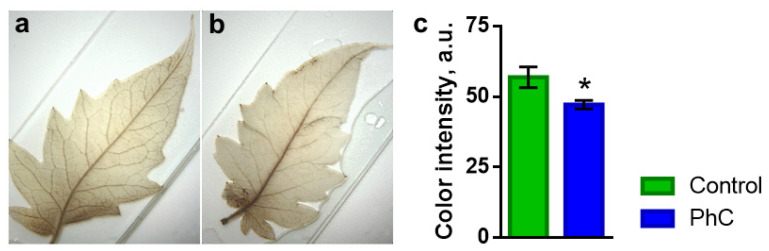
Influence of photoconversion coatings on the content of H_2_O_2_ in the leaves of tomato plants: (**a**,**b**) photograph of leaves of a control tomato plant and a plant grown under a photoconversion coating, stained with DAB; (**c**) relative content of H_2_O_2_ in the leaves of control plants and plants grown under a photoconversion coating. *: *p* < 0.05.

The reactive oxygen species (ROS) content was estimated by the most long-lived form, hydrogen peroxide. The amount of H_2_O_2_ was determined by DAB staining (Figure 7a,b). A decrease in the level of H_2_O_2_ was found in plants grown under photoconversion films. In the leaves of such plants, the content of H_2_O_2_ was lower than the control values by 17 ± 3% (Figure 7c).

## 4. Discussion

Plants were grown under photoconversion films, which produce photoconversion of UV-A radiation and violet light into blue and red light [10]. The use of a photoconversion coating increases the size of the leaves (Figure 2). The reason for this may be the experimentally observed stimulation of the process of photosynthesis. It was shown that in plants grown under a photoconversion coating, there is an increase in CO_2_ assimilation—an integral indicator of photosynthesis activity (Figure 3b). Assimilation, as a rule, is closely related to the level of transpiration: transpiration reflects the degree of stomatal openness and, consequently, the availability of CO_2_ for cells. In plants grown under a photoconversion coating, assimilation increases against the background of constant transpiration and intercellular CO_2_ concentration (Figure 3b–d). This indicates the predominant regulation at the level of the activity of photosynthetic processes, rather than the availability of CO_2_. This is confirmed by the stimulation of the light-driven reactions of photosynthesis, found when using a photoconversion coating. Such stimulation is evidenced by an increase in the Φ_PSII_ level, which reflects the quantum yield of Photosystem 2, and a decrease in the NPQ level, reflecting non-photochemical quenching of fluorescence (Figure 3f).

Photoconversion coatings can affect photosynthesis in two ways: (1) by increasing photosynthetically active radiation (PAR) and (2) by reducing the UV component in the spectrum. The coatings used in this work have photoluminescence maxima at 450 and 630 nm, which quite well correspond to the absorption maxima of tomato plants, located in the region of 440 and 675 nm [29]. Stimulating photosynthesis by increasing the proportion of light in the blue and red regions of the spectrum has been shown in a large number of works [1,2,3,4,5,6,10,11,14]. Such stimulation can be caused both by a direct increase in the amount of energy available for photosynthesis and by the adjustment of physiological processes due to signaling pathways starting from photoreceptors. The first is unlikely due to the small value of the increase in PAR (Figure 1), which suggests the role of regulatory systems. Currently, it is known that all plants have receptors for red and far-red light—phytochromes (phy)—blue and UV-A light—cryptochromes, phototropins and members of the Zeitlupe family (cry, phot, ZTL, FKF1 and LKP2)—as well as UV-B light—UV RESISTANCE LOCUS 8 (UVR8) [30,31]. Changes in the intensity of light in these wavelength regions lead to morphometric and physiological rearrangements, which is known as photomorphogenesis. Due to the intersection of signaling pathways from different photoreceptors, the amplification of light at different wavelengths can lead to similar results. Thus, both blue and red light, through the pathways of phototropins and phytochromes, cause leaf expansion [32,33], which was shown in plants grown under a photoconversion coating (Figure 2). During the regulation of photosynthetic processes, red light through the signaling pathways of phytochromes activates chlorophyll synthesis and regulates the number of chloroplasts [34] and blue light through the phototropin cascade affects the position of chloroplasts in the cell [35]. In sum, this can also contribute to the stimulation of photosynthesis observed in our experiments (Figure 3). For other effects, the ratio of the intensity of the luminous flux at certain wavelengths plays a decisive role. Thus, a low ratio of red (660 nm, an activated form of phytochrome) to far-red (730 nm, an inactivated form of phytochrome) light promotes increased NADPH oxidase-induced generation of H_2_O_2_, which can damage components of the photosynthetic apparatus [36,37,38]. At a high red/far-red light ratio, on the contrary, the level of H_2_O_2_ decreases due to an increase in antioxidant protection as a result of the induction of the transcriptional activity of some genes of antioxidant enzymes [39]. The photoconversion coatings used in our experiments have a photoluminescence maximum at 630 nm [10], which leads to an increase in the red/far-red light ratio. This causes a decrease in the content of H_2_O_2_ in plants grown using photoconversion coatings (Figure 7).

Partial exclusion of UV radiation from the spectrum can also have a significant biological effect. Unlike UV-B (280–315 nm) and UV-C (100–280 nm) radiation, the UV-A (315–400 nm) used in our experiments is usually not associated with a pronounced inhibitory effect on morphometric parameters, but it has traditionally been considered to be damaging for photosynthesis [40,41]. The damaging effect of UV-A on the catalytic Mn cluster of a water-oxidizing complex, protein D1 and D2 subunits of the PSII reaction center, QA and QB binding sites and Rubisco has been described [41,42,43,44,45]. This corresponds to lower values of indicators of both light-driven and light-independent reactions of photosynthesis, recorded in our experiments in control plants that received a greater amount of UV-A radiation (Figure 3). An increase in the level of ROS, which occurs mainly due to the work of cytoplasmic NADPH oxidase, has been shown to be one of the key mechanisms of action of UV radiation, including the UV-A component [44,46,47]. Thus, under conditions of a decrease in the UV-A spectrum, a decrease in the activity of NADPH oxidase contributes to a decrease in the content of H_2_O_2_ in plants grown under a photoconversion coating (Figure 7), which is in line with other studies with a decrease in the UV-A spectrum [47].

A decrease in the ROS level can also explain another effect found in plants grown under the photoconversion coating—a decrease in resistance to heat stress (Figure 4). It is now well known that ROS and, in particular, their most long-lived form, H_2_O_2_, not only causes damage to macromolecules, but also performs signaling functions [20,48,49]. H_2_O_2_ is involved in switching between modes of increased productivity and increased resistance due to regulation at the physiological and genetic levels [20,50]. One of the most significant mechanisms of such a switch is the redistribution of energy resources between the processes of biosynthesis—productivity and the antioxidant system protection. The reduced capacity of the antioxidant system may contribute to the reduced resistance of plants grown under a photoconversion film to heat stress found in our experiments (Figure 4). It should also be noted that H_2_O_2_ is a component of distant stress signals. Long-distance electrical signals are jointly propagating and mutually reinforcing waves of ROS, Ca^2+^ and electric potential [16,51]. In our previous work, we showed that in plants grown under a photoconversion coating, the amplitude and the conductivity of long-distance electrical signals decreases [10].

The spread of ES causes a rearrangement of physiological processes, which contributes to the formation of resistance (systemic acquired acclimation, SAA) to stressors, including heat stress [17,18,22]. Functional response is formed due to shifts in ionic concentrations (primarily Ca^2+^ and H^+^) accompanying signal propagation [18,52,53]. These functional responses include signal-induced inhibition of photosynthesis (Figure 5), which results in reduced ROS generation at ETC and thus reduced damage to the photosynthetic apparatus under suboptimal conditions. Signal-induced reduction in transpiration (Figure 5) prevents excess water loss and plant wilting. In addition, it is necessary to emphasize the recently demonstrated [54] ability of ES to increase the expression of the heat shock factor (HSF) families which promote the synthesis of heat shock proteins (HSP), which are directly involved in heat stress protection. The protective effect of local heating-induced ES against tomato plants subjected to heat stress is also shown in our experiments (Figure 6). One of the key factors in increasing resistance as a result of ES, a temporarily inhibited photosynthesis, occurs both directly and indirectly, through hormonal shifts that contribute to stomatal closure and a decrease in CO_2_ availability [55,56,57,58]. The absence of a significant effect on transpiration, by which the stomata openness is judged (Figure 6), suggests the development of the effect in tomato plants mainly along the path of direct influence on photosynthetic processes. Since the amplitude of ion shifts, as a rule, correlates with the amplitude of ES [23,59], the weakening of signal-induced resistance in plants grown using photoconversion technologies (Figure 6) can be explained by the suppression of stress signals.

## 5. Conclusions

Photoconversion coatings for greenhouses increase the size of tomato plants and activate the process of photosynthesis. Due to the change in the quality of light and a related possible decrease in the content of ROS in tissues in such plants, the development program is probably shifted towards productivity to the detriment of protective functions. Plants grown using photoconversion technologies have reduced resistance to heat stress as well as less pronounced regulation of resistance by long-distance electrical signals. Thus, when using photoconversion technologies, it is necessary to pay increased attention to maintaining stable conditions during plant cultivation. The development of specific methods for improving practical greenhouse plant production requires additional experimental research, including with various options for lighting sources that closely simulate both sunlight and light sources traditionally used in greenhouses.

## Data Availability

Data is contained within the article and supplementary material.

## Supplementary Materials

The following are available online at https://www.mdpi.com/article/10.3390/plants11020229/s1, Figure S1: Photoluminescence spectrum of a fluoropolymer film containing fluorophores and gold nanoparticles (solid line) and without photoconverting components (dotted line), obtained using a xenon reference light source.
