# Supplementary material for: Effect of Photoconversion Coatings for Greenhouses on Electrical Signal-Induced Resistance to Heat Stress of Tomato Plants
Source: Plants (Basel). 2022 Jan 17;11(2):229. doi: 10.3390/plants11020229 (PMC8779642; doi:10.3390/plants11020229)
Supplement: Supplementary file 1 [file plants-11-00229-s001.zip › plants-1527962-supplementary.pdf]

Supplementary Materials

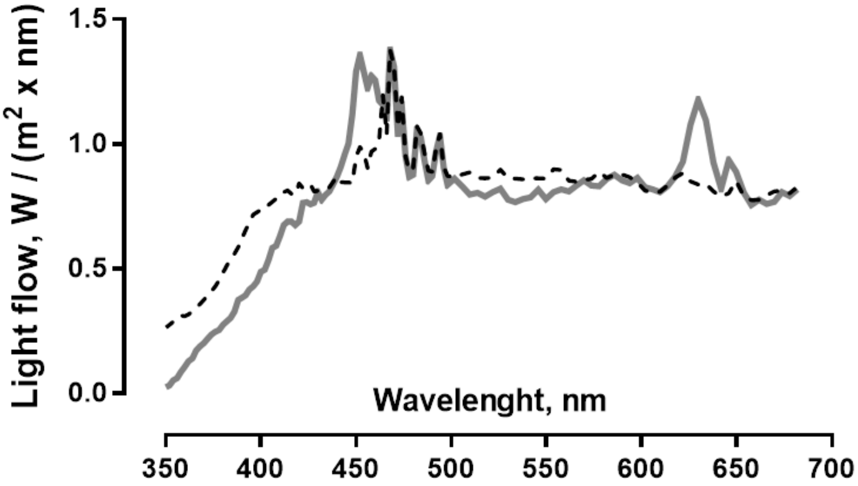

Figure S1. Photoluminescence spectrum of a fluoropolymer film containing fluorophores and gold nanoparticles (solid line) and without photoconverting components (dotted line), obtained using a xenon reference light source.
